# Primary Succession of Nitrogen Cycling Microbial Communities Along the Deglaciated Forelands of Tianshan Mountain, China

**DOI:** 10.3389/fmicb.2016.01353

**Published:** 2016-08-30

**Authors:** Jun Zeng, Kai Lou, Cui-Jing Zhang, Jun-Tao Wang, Hang-Wei Hu, Ju-Pei Shen, Li-Mei Zhang, Li-Li Han, Tao Zhang, Qin Lin, Phillip M. Chalk, Ji-Zheng He

**Affiliations:** ^1^State Key Laboratory of Urban and Regional Ecology, Research Center for Eco-Environmental Sciences, Chinese Academy of SciencesBeijing, China; ^2^College of Resources and Environment, University of Chinese Academy of SciencesBeijing, China; ^3^Institute of Applied Microbiology, Xinjiang Academy of Agricultural SciencesUrumqi, China; ^4^Faculty of Veterinary and Agricultural Sciences, The University of Melbourne, MelbourneVIC, Australia

**Keywords:** N cycling microbial community, glacier foreland, soil carbon and nitrogen, primary succession, Tianshan Mountain

## Abstract

Structural succession and its driving factors for nitrogen (N) cycling microbial communities during the early stages of soil development (0–44 years) were studied along a chronosequence in the glacial forelands of the Tianshan Mountain No.1 glacier in the arid and semi-arid region of central Asia. We assessed the abundance and population of functional genes affiliated with N-fixation (*nifH*), nitrification (bacterial and archaeal *amoA*), and denitrification (*nirK*/*S* and *nosZ*) in a glacier foreland using molecular methods. The abundance of functional genes significantly increased with soil development. N cycling community compositions were also significantly shifted within 44 years and were structured by successional age. Cyanobacterial *nifH* gene sequences were the most dominant N fixing bacteria and its relative abundance increased from 56.8–93.2% along the chronosequence. Ammonia-oxidizing communities shifted from the *Nitrososphaera* cluster (AOA-*amoA*) and the *Nitrosospira* cluster ME (AOB-*aomA*) in younger soils (0 and 5 years) to communities dominated by soil and sediment 1 (AOA-*amoA*) and *Nitrosospira* Cluster 2 Related (AOB-*aomA*) in older soils (≥17 years). Most of the denitrifers closest relatives were potential aerobic denitrifying bacteria, and some other types of denitrifying bacteria (like autotrophic nitrate-reducing, sulfide-oxidizing bacteria and denitrifying phosphorus removing bacteria) were also detected in all soil samples. The regression analysis showed that N cycling microbial communities were dominant in younger soils (0–5 years) and significantly correlated with soil total carbon, while communities that were most abundant in older soils were significantly correlated with soil total nitrogen. These results suggested that the shift of soil C and N contents during the glacial retreat significantly influenced the abundance, composition and diversity of N cycling microbial communities.

## Introduction

Global warming has caused shrinkage of many alpine mountain glaciers in the world over the last century, and the rate of retreat of most glaciers appears to have accelerated in recent decades (Li et al., 2011; Wang et al., 2011). As glacial retreat occurs, terrestrial habitats are exposed and the barren land experiences a succession of soil processes, including carbon (C) and nitrogen (N) accumulation, transformation and nutrient cycling (Hopkins et al., 2007; Nemergut et al., 2007). Of these, microbially mediated N transformation processes are one of the basic biogeochemical processes in these forelands. Nitrogen exists naturally in dinitrogen gas or organic compounds, both of which cannot be directly used by plants. Only some specialized microorganisms, like N_2_-fixing communities and microorganisms involved in organic compound mineralization, are able to transform N_2_ or organic compounds into ammonia. Of these, N_2_-fixation is a major source of N inputs to many natural ecosystems, especially in newly exposed glacial foreland ecosystems (Yeager et al., 2004; Nemergut et al., 2007; Abed et al., 2010; Göransson et al., 2011). Ammonia is first oxidized to nitrite by ammonia-oxidizing prokaryotes through nitrification, which is the first and rate limiting step for N cycling in soil. This transformation is followed by the denitrification process that is mediated by nitrate reducers and denitrifiers; nitrate can then be stepwise reduced to nitrite or further to dinitrogen gas. Therefore, at the early stage of soil development the colonization of N cycling microorganisms are pivotal to the whole ecosystem functioning (Ollivier et al., 2011).

However, few studies have investigated the N cycling microbial communities in the retreated glacier forelands during the soil successional stages. Nemergut et al. (2007) found that the relative abundance of N_2_-fixing cyanobacteria significantly increased along a chronosequence in the unvegetated, early successional degalaciated forelands in southeastern Peru. Duc et al. (2009) revealed that free-living diazotrophic diversity in the alpine glacier bulk soils increased with soil age, but their diversity decreased with the presence of pioneer plants. Deiglmayr et al. (2006) showed that actinobacteria were the most dominant nitrate-reducing community and its abundance increased with soil age in an alpine glacier. Besides, Kandeler et al. (2006) and Brankatschk et al. (2011) revealed that the relative abundance of denitrifying bacteria (*narG* and *nirS*) and nitrification microorganisms (AOA and AOB) were high in the initial stage of soil development. These investigations were mainly focused on certain groups of the nitrogen cycling microbial communities. We still lack a comprehensive understanding of the microbial communities involving in the N cycling processes over the deglaciated time and the principal factors driving these changes (Ollivier et al., 2011).

Nitrogen is usually the main limiting factor in primary successional ecosystems and is crucially important for further soil development (Göransson et al., 2011). Ollivier et al. (2011) divided ecosystem chronosequences into initial, intermediate and mature phases based on the total N concentrations above 0.1, 0.2, and 0.7%, respectively. In the initial stage, N_2_ fixing microorganisms may be the most diverse and active community of the N cycling microbial communities. Their colonization not only increased the N input but also contributed to soil stabilization. Besides, due to the lack of vegetation, there is limited competition for N resources resulting in a sufficiency of ammonia to stimulate the nitrification process and the development of nitrifying organisms. In the intermediate stage, the increasing plant coverage results in an increasing C input. In this stage, plant and microbial communities may compete for N that result in increased N_2_-fixation activity in the rhizosphere. Hence, the intermediate nutrient level may cause the highest microbial diversification. In the mature stage, soils are well developed and densely covered with vegetation so that nitrification and denitrification become highly dominant microbial processes. While this concept was informative, more research is needed for validation.

In this paper, we chose Tianshan Mountain No.1 glacier forelands to investigate the succession of N_2_-fixing, nitrification and denitrification communities along recently deglaciated forelands. Previous research has shown that the glacier is highly sensitive to climate change (change of temperature and precipitation), where the total glacial area has reduced by 11.5% in the past 50 years (Li et al., 2011, 2013; Wang et al., 2011). Although the glacier forelands have gone through 50 years of succession, the soil is still devoid of vegetation. Thus, we collected unvegetated samples representing soils ice free for 0, 5, 17, 31, and 44 years and an alpine meadow (mature) soil (>1500 years deglaciated, used as a reference, Ref). We used qPCR to quantify N_2_-fixing microorganism (*nifH* gene), nitrification (*amoA* genes of ammonia oxidizing archaea and bacteria) and denitrification (*nirS, nirK* and *nosZ*) communities along the glacier foreland chronosequence. Terminal restriction length polymorphism (T-RFLP) combined with clone library methods were used to study the functional compositions of the microbial communities.

## Materials and Methods

### Site Description and Soil Sampling

**FIGURE 1:**
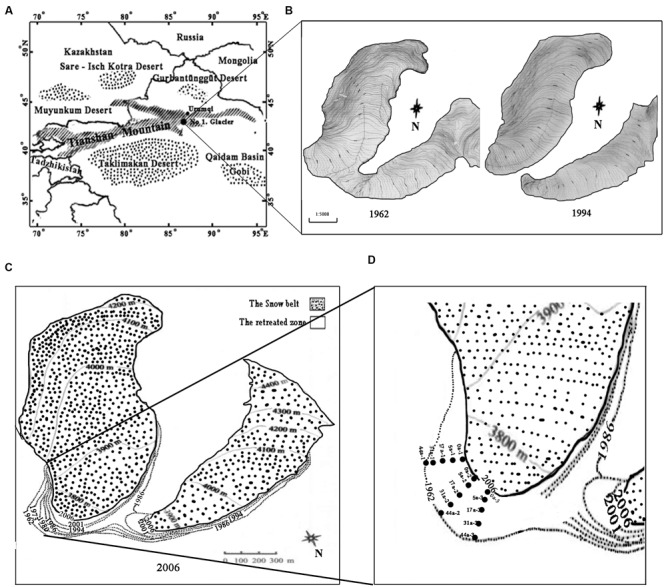
**Map of the sampling sites on the east branch of the Tianshan Mountain Glacier No. 1 as modified after Li et al. (2011). (A)** Location of the Glacier No.1; **(B)** Changes in Glacier No. 1 during the period of 1962–1994; **(C)** The boundaries of glacier No. 1 in different periods (dashed lines represent the glacial boundaries from 1962 to 2001, and the solid line represents the glacial boundary in 2006); **(D)** Five transect sampling sites are denoted by the dotted rectangles.

Tianshan Mountain No.1 glacier is a small cirque-valley glacier, located at the headwaters of the Urumqi River, western Tianshan Mountain, Xinjiang, China (43°06′N, 86°49′E). It is about 3,840 m above sea level and is surrounded by deserts (**Figure 1A**). The annual mean temperature is -5.2°C, with the negative temperature prolonged for 7–8 months with the lowest temperature for January at -15.6°C and the highest temperature 4.9°C (in July). The average annual precipitation is 645.8 mm (Xu et al., 2011). The observation of Glacier No.1 started in 1959 and more than 50 years’ data showed that the air temperature has been continuously rising since 1985, and the rate has accelerated since 1995. From 1997 up to the present, the average temperature has increased by 1°C, and the glacier has split into two separate parts since the end of 1993 (**Figure 1B**) (Li et al., 2013). From 1959 to 1993, the glacier retreated at an average rate of 4.5 m⋅year^-1^, while the retreating rate was slower in the east branch (3.5 m⋅year^-1^) but faster in the west branch (5.8 m⋅year^-1^) from 1993 to 2004 (Xu et al., 2011; Li et al., 2011, 2013).

In this study, we selected only the well documented east branch for investigation. In August 2008 we set up five transects that ran parallel along the direction of the glacial retreat. Sampling sites were located at 0, 18, 60, 120, and 180 m from the glacier terminus, representing the successional ages of 0, 5, 17 (a glacier mean retreating rate of 3.5 m⋅year^-1^), 31 and 44 years (4.5 m⋅year^-1^), respectively (**Figures 1C,D**) (Li et al., 2013). The deglaciation time of each soil site was inferred from the database compiled by the Tianshan Glaciological Station of the Chinese Academy of Sciences. Samples of the top 5 cm of soil were collected (three replicates for each site) with a small spade at an interval of 15–20 m along each transect. In addition, an alpine meadow soil about 500 m away from the glacier terminus was selected as a control (the reference soil, Ref). The collected soil samples were stored in an ice box and immediately transported to the laboratory. The samples were divided into two sub-samples: one was freeze-dried and stored at -80°C, and another was stored at 4°C for biogeochemical analyses.

### Soil Chemical Properties

Soil pH was determined in 0.01 M CaCl_2_ solution and measured by a glass electrode using a Mettler DL-25 pH meter. The total nitrogen (TN) and carbon (TC) contents were determined by dry combustion at 1200°C on an elemental analyzer (LECO CNS 2000) with infrared and thermal conductivity detectors, respectively. Total phosphorus (TP) was measured by the perchloric acid-sulfate method (Tscherko et al., 2003). Available P (AP) was colorimetrically determined after acetate–lactate extraction (Tscherko et al., 2003). Nitrate (NO_3_^-^-N) and ammonium (NH_4_^+^-N) were extracted with 2 M KCl and measured using a LACHAT Quickchem Automated Elemental Analyzer.

### DNA Extraction and Polymerase Chain Reaction (PCR)

The soil DNA was extracted from triplicate samples (0.5 g) using a Power soil DNA isolation kit (MO BIO Laboratories, San Diego, CA, USA) according to the manufacturer’s protocol. The quantity of the extracted DNA was checked with NanDrop ND-2000 UV–vis Spectrophotometers (NanoDrop Technologies, USA).

Quantitative PCR (qPCR) assay was carried out with a Bio-Rad iQTM 5 Thermocycler (Bio-Rad Laboratories, Hercules, CA, USA) using SYBR green as the detection system in a reaction mixture of 25 μl containing 0.5 μM of each primer, 12.5 μl of SYBR green PCR master mix (TaKaRa, Japan), and 1 μl of fivefold diluted template DNA (∼10 ng). Standard curves for the six gene qPCR assays were developed as described by He et al. (2007). Briefly, N cycling functional genes (*nifH, amoA, nirK/S* and *nosZ*) were PCR amplified from the fivefold diluted environmental DNA with the primers listed in **Table 1**, and then the PCR products were purified and cloned (see below the clone and sequencing). Plasmids used as standards for quantitative analyses were extracted from the correct insert clones of each targeted gene. The concentration of plasmid DNA was determined on the Nanodrop^®^ ND-2000 UV–vis Spectrophotometer, and the copy numbers of target genes were calculated. Ten-fold serial dilutions of a known copy number of the plasmid DNA were subjected to qPCR assay in triplicate to generate an external standard curve. A dissociation curve was included at the end of the qPCR program to evaluate potential primer–dimers and non-specific amplification products. PCR for terminal restriction fragment length polymorphism analysis (T-RFLP) and clone library construction were carried out with Eppendorf Mastercycler (Eppendorf, Germany). PCR reaction mixtures (50 μl total volume) contained 25 μl of 2× DNA reaction buffer (Premix Taq 1.5 U, 3 mM MgCl_2_, 200 μM dNTP), 1.5 μl of fivefold diluted template DNA (∼10 ng), 0.25 μl of each primer (10 μM), 23 μl of ddH_2_O. PCR conditions and primers used are summarized in **Table 1**. After amplification, the PCR products were checked for size and specificity by electrophoresis on 1% (w/v) agarose gel and fragment sizes were estimated using a 100 bp marker ladder (Invitrogen, USA).

**Table 1:** List of PCR primers used in this study for T-RFLP and sequencing analyses.

Target gene	Primer set	Sequences (5′-3′)	qPCR efficiency	Thermal profile	Reference
*amoA* (AOA)	CrenamoA23f^a^	ATGGTCTGGCTWAGACG	87%	qPCR: 95°C/1 min; 35 cycles of 95°C/30 s, 55°C/30 s,72°C/30 s.	Avrahami and Conrad, 2003
	CrenamoA616r	GCCATCCATCTGTATGTCCA		T-RFLP: 94°C/5 min; 10 cycles of 94°C/30 s, 60°C/30 s (-0.5°C/cycle), 72°C/30 s; 25 cycles of 94°C/30 s, 55°C/45 s, 72°C/1 min, 72°C/5 min.	
*amoA* (AOB)	amoA1F^a^	GGGGTTTCTACTGGTGGT	78%	qPCR: 95°C/1 min; 35 cycles of 95°C/30 s, 60°C/30 s,72°C/30 s.	Rotthauwe et al., 1997
	amoA2R	CCCCTCKGSAAAGCCTTCTTC		T-RFLP: 94°C/5 min; 10 cycles of 94°C/30 s, 62°C/45 s (-0.5°C/cycle), 72°C/1 min; 25 cycles of 94°C/30 s, 57°C/45 s, 72°C/1 min, 72°C/5 min.	
*nirS*	Nirs-cd3aF^a^	GTSAACGTSAAGGARACSGG	97%	qPCR: 94°C/2 min; 5 cycles of 94°C/1 min, 58°C/1 min (-1°C/cycle), 72°C/30 s; 30 cycles of 94°C/30 s, 53°C/1 min, 72°C/30 s.	Nirs-cd3aF: Michotey et al., 2000
	Nirs-R3cd	GASTTCGGRTGSGTCTTGA		T-RFLP: 95°C/5 min; 6 cycles of 95°C/15 s, 63°C/30 s (-1°C/cycle), 72°C/30 s; 25 cycles of 95°C/15 s, 58°C/30 s, 72°C/30 s, 72°C/5 min.	Nirs-R3cd: Throbäck et al., 2004
*nirK*	nirK-F1aCu^a^	ATCATGGTSCTGCCGCG	95%	qPCR: 95°C/3 min; 6 cycles of 95°C/30 s, 63°C/30 s (-1°C/cycle), 72°C/30 s; 30 cycles of 95°C/30 s, 58°C/30 s, 72°C/30 s.	Throbäck et al., 2004
	nirK-R3Cu	GCCTCGATCAGRTTGTGGTT		T-RFLP: 95°C/5 min; 6 cycles of 95°C/30 s, 63°C/30 s (-1°C/cycle), 72°C/20 s; 25 cycles of 95°C/30 s, 58°C/30 s, 72°C/30 s, 72°C/5 min.	
*nosZ*	nosZ2F	CGCRACGGCAASAAGGTSMSSGT	97%	qPCR: 95°C/1 min; 6 cycles of 95°C/30 s, 65°C/30 s (-1°C/cycle), 72 °C/30 s; 30 cycles of 95°C/30 s, 60°C/30 s, 72°C/30 s.	Henry et al., 2006
	nosZ2R^b^	CAKRTGCAKSGCRTGGCAGAA		T-RFLP b: 95°C/5 min; 30 cycles of 95°C/30 s, 60°C/30 s, 72 °C/30 s, 72°C/5 min.	
	nosZ-F^a^	CGYTGTTCMTCGACAGCCAG			
*nifH*	IGK3	GCIWTHTAYGGIAARGGIGGIATHGGIAA	96%	qPCR:95°C/5 min; 40 cycles of 95°C/30 s, 58°C/30 s, 72°C/30 s; 72°C/5 min.	Gaby and Buckley, 2012
	DVV	ATIGCRAAICCICCRCAIACIACRTC			

*^*a*^(6-FAM) was attached to the 5′ end of the primers. ^*b*^(nosZ-F + nosZ2R) were used for nosZ gene T-RFLP analysis.*

### T-RFLP Analysis

The FAM-labeled PCR products were digested with *Rsa I* (AOA-*amoA* and *nosZ*), *Hha* I (AOB-*amoA*), and *Hae* III (*nirS* and *nirK*) in 20 μl reactions. All the restriction enzymes were purchased from TaKaRa (TaKaRa, Dalian, China). The three replicates of PCR products were mixed in equal ratio and purified using a Wizard SV Gel and PCR Clean-up system (Promega, San Luis Obispo, CA, USA). The enzyme reaction mixture (20 μl) contained 2 μl of 10× Buffer, 0.5 μl of restriction enzyme (10 U⋅μl^-1^, TaKaRa), <1 μg of amplicon and ddH_2_O. All the digests were incubated at 37°C for 3 h, followed by deactivation at 95°C for 15 min. The T-RFLP profiles of each digest were determined by ABI3700 (Applied Biosystems, USA).

### Cloning and Sequencing

Due to the sites of *nifH* gene primer pairs contained some inosine which could affect the FAM, we only used the cloning method for the investigation of the N_2_ fixation community, while other genes were detected by both T-RFLP and cloning methods. PCR amplifications for the *nifH* gene clone libraries construction were performed on the three replicate DNA extracts of each site, which were then pooled for cloning. Two samples (0 year and Ref) were selected for *amoA* gene (AOA and AOB) clone library construction. Samples from 31 years and Ref were used for *nirS* and *nirK* genes clone libraries construction. Three clone libraries were constructed for *nosZ* gene from samples 0, 31 years and Ref, respectively. The purified non-FAM-labeled PCR products were ligated into a pMD19-T Vector and transformed into *E. coli* JM109 competent cells (TaKaRa, Dalian, China). The transformed cells were plated on Luria-Bertani plates containing 100 μg⋅ml^-1^ of ampicillin, and incubated for 12–16 h at 37°C. In order to precisely find the T-RFs determined from environmental samples, each gene positive clone was re-amplified with their FAM-labeled primer sets and detected according to the above T-RFLP procedure. The positive clones that had the same T-RFs with environmental samples were sequenced by the Sanger sequencing method.

### Phylogenetic Analysis and Genbank Accession Numbers

All the functional gene sequences obtained in this study were submitted for comparison to the Genbank database using the BLAST. Neighbor-joining phylogenetic trees were constructed from dissimilar distance and pairwise comparisons with the Jukes–Cantor distance model using the MEGA software (version 6.0). Bootstrap value of 1000 replications was assessed in the analysis. Each gene sequences obtained from this study has been deposited in the Genbank database under accession numbers: KM043896 to KM044003 (*nifH*); KJ660894 to KJ660921 (AOA); KJ660862 to KJ660893 (AOB); KJ145298 to KJ145319 (*nirS*); KJ660922 to KJ660951 (*nirK*), KJ145248 to KJ145297 (*nosZ*).

### Statistical Analysis

The relative abundance of a T-RF was calculated by dividing the peak height of the T-RF by the total peak height of all T-RFs in the profile. The peaks with heights <1% of the total peak height were not included for further analyses (Cao et al., 2012). Shannon index and Bray–Curtis index were calculated by using QIIME 1.7.0 (Caporaso et al., 2010). Spearman’s correlation analysis was conducted to examine the relationships between soil chemical properties and functional gene abundances and community relative abundances in SPSS 21.0 (IBM Co., Armonk, NY, USA). Correlation best-of-fit model for the functional communities’ relative abundance and soil C and N was carried out by curve estimation in SPSS. Multiple regression analysis was applied to evaluate the relationship between soil chemical properties (TC, TN, TP, AP and soil age) and the abundance of those functional genes. The significance of the community composition difference was tested by the permutation multivariate analysis of variance (PERMANOVA) in R 3.1.2 using the Vegan package (Jimenez et al., 2011). The Mantel test was performed to examine the relationships between soil chemical properties and functional community compositions.

## Results

### Soil Physicochemical Properties

Chemical parameters of the glacier foreland soils varied considerably along the chronosequence (**Table 2**). Spearman’s correlation analysis showed that soil total carbon (TC, *r* = 0.86, *p* < 0.01), available phosphorus (AP, *r* = 0.59, *p* < 0.05), pH (*r* = 0.61, *p* < 0.05), ammonium (NH_4_^+^, *r* = 0.88, *p* < 0.01) and nitrate (NO_3_^-^, *r* = 0.63, *p* < 0.01) were significantly and positively correlated with the successional age.

**Table 2:** Soil properties of the successional sites across the Tianshan Mountain Glacier No.1

Soil properties		Successional age					
		0 a	5 a	17 a	31 a	44 a	Ref
pH		7.57 ± 0.18a	7.68 ± 0.12a	7.58 ± 0.15a	7.73 ± 0.09a	7.88 ± 0.09a	7.30 ± 0.08b
TC	(g⋅kg^-1^)	1.56 ± 0.08d	2.15 ± 0.05c	2.61 ± 0.04bc	2.54 ± 0.27bc	2.91 ± 0.12b	14.3 ± 0.05a
TN	(g⋅kg^-1^)	0.17 ± 0.05e	0.31 ± 0.08b	0.29 ± 0.04c	0.14 ± 0.04f	0.21 ± 0.02d	1.11 ± 0.40a
TP	(g⋅kg^-1^)	0.67 ± 0.10a	0.92 ± 0.12a	0.73 ± 0.04a	0.73 ± 0.08a	1.08 ± 0.10a	0.99 ± 0.09a
AP	(mg⋅kg^-1^)	4.30 ± 0.26d	4.80 ± 0.11d	3.90 ± 0.12d	13.04 ± 1.15b	10.21 ± 1.00c	16.63 ± 0.85a
NH_4_^+^	(mg⋅kg^-1^)	0.97 ± 0.01e	0.98 ± 0.00e	1.34 ± 0.00b	1.31 ± 0.01c	1.78 ± 0.03a	1.26 ± 0.02d
NO_3_^-^	(mg⋅kg^-1^)	0.11 ± 0.01c	0.16 ± 0.02bc	0.06 ± 0.04c	0.23 ± 0.06b	0.27 ± 0.08b	1.92 ± 0.08a

*TC, soil total carbion; TN, soil total nitrogen; TP, soil total phosphorus; AP, avaiabliable phosphorus. a–e: Significant differences as revealed by one-way ANOVA on ranks (*p* < 0.05) are indicated by different letters.*

### Abundance and Community Composition of the N Cycling Microbial Communities

**FIGURE 2:**
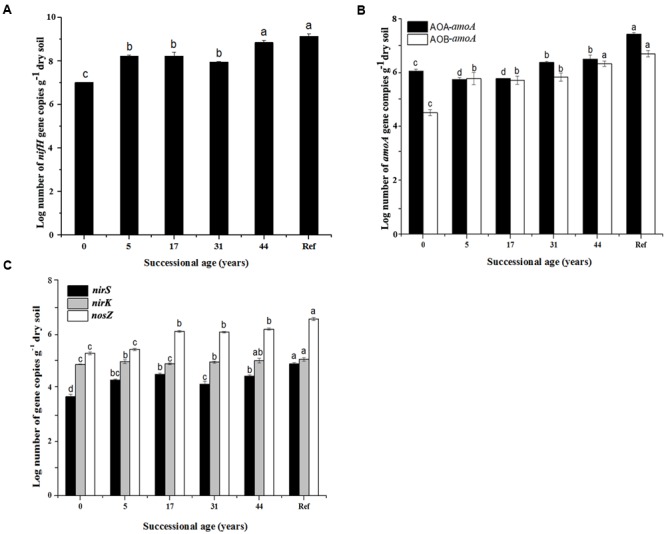
**Gene copy numbers of the major steps of nitrogen cycling functional genes (*nifH*, AOA-*amoA*, AOB-*amoA, nirS, nirK* and *nosZ*). (A)** *nifH* gene abundance; **(B)** *amoA* gene abundance; **(C)** *nirS, nirK* and *nosZ* genes abundance; (*n* = 3, error bars represent standard error of means). Only significant differences as revealed by one-way ANOVA on ranks (*p* < 0.05) are indicated by different letters.

The numbers of N cycling community genes (*nifH*, AOA*-amoA*, AOB*-amoA, nirK, nirS* and *nosZ*) generally presented an increasing trend along the time sequences. They showed significant increase in the younger (0 and 5 years) and the older soils (44 years), but were relatively stable during 17–31 years (**Figure 2**). The lowest *nifH* gene copy numbers was in 0 year (3.3 × 10^7^ copies g^-1^ dry soil) and increased up to 3.6 × 10^8^ in 44 years (**Figure 2A**). The AOA abundance was higher than AOB in most soil samples. The AOA-*amoA* gene copy numbers significantly decreased from 3.55 × 10^6^ at 0 year to 2.15 × 10^6^ copies g^-1^ dry soil at 17 years, but significantly increased after 31 years. Generally, AOB-*amoA* gene copy numbers ranged from 2.5 × 10^4^ to 5.75 × 10^6^ copies g^-1^ dry soil (**Figure 2B**). The *nirK* and *nirS* genes were tenfold less abundant than *nosZ* gene: *nirK* ranged from 4.5 × 10^3^ to 6.23 × 10^3^ copies g^-1^ dry soil, while *nirS* ranged from 5.2 × 10^2^ to 4.4 × 10^3^ (**Figure 2C**).

**FIGURE 3:**
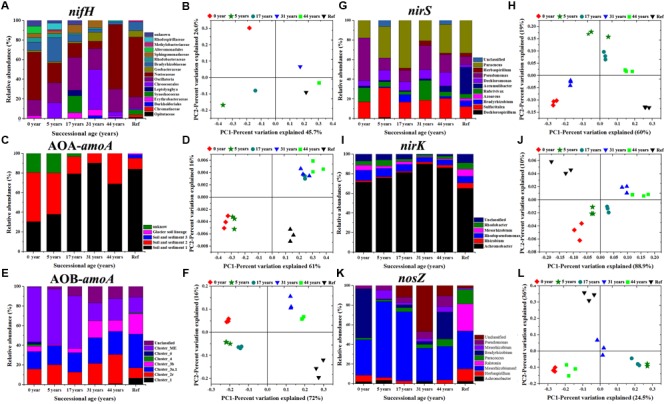
**Microbial community composition and relative abundance of N-fixing bacteria based on *nifH* gene **(A)**, ammonia-oxidizing archaea and bacteria based on AOA-*amoA***(C)** and AOB-*amoA* genes **(E)**, denitrifying bacteria based on *nirS***(G)**, *nirK***(I)** and *nosZ***(K)** genes.** Principal coordination analysis (PCoA) derived from the Bray-Curtis dissimilarity matrices are based on the relative abundance of *nifH* **(B)**, AOA-*amoA* **(D)**, AOB-*amoA* **(F)**, *nirS* **(H)**, *nirK* **(J)** and *nosZ* genes **(L)**, respectively.

A clear succession of N cycling functional communities across the five successional sites of the glacier forelands is shown in **Figure 3**. The *nifH* gene library was composed of 480 positive clones (80 clones per sample) representing 4 phyla (Cyanobacteria, Proteobacteria (α-, β-, γ-, and δ-), Firmicutes, Verrucomicrobia) and unclassified groups (**Figure 3A**; Supplementary Figure S1). Cyanobacterial *nifH* sequences were dominant in the six soil samples and its relative abundance increased from 56.8% in 5 years to 93.2% in 44 years. The family Nostocaceae was the dominant group in 0 year (accounted for 48.5%) and gradually declined to 7.6% in 31 years, but then dramatically increased in the samples 44 a (65.8%) and Ref (61%). The phylum Proteobacteria was the most diverse group that contained 4 classes (α-, β-, γ-, and δ-). The α-proteobacteria class was the most diverse and abundant in the phylum. Their relative abundance in the class decreased from 39% in the sample 5 years to 5.5% in the Ref. The T-RFLP analysis of AOA-*amoA* gene by the *RsaI* enzyme yielded 14 major distinct terminal restriction fragments (TRFs) across all the samples. Cloning and sequencing analysis and phylogenetic analysis could assign the 14 T-RFs into four clusters: Soil and Sediment 1 (T-RFs 56, 58, 60, 61, 198, 471, 566, 568, and 570 bp), Soil and Sediment 2 (T-RFs 17, 18, and 279 bp), Soil and Sediment 3 (T-RF 298 bp), Glacial soil lineage (T-RF 256 bp) and unknown group (**Figure 3C**; Supplementary Figure S2). Soil and Sediment 2 was the dominant group in the younger soils (0 and 5 years) which accounted for about 50%. Its preeminence was replaced by Soil and Sediment 1 in older soils (17–44 years) and Ref, which accounted for over 80%. Soil and Sediment 3 and Glacial soil lineage groups were only detected in the developed stage (**Figure 3C**). In total, 13 major AOB-*amoA* T-RFs were detected in all soil samples, which divided into: Cluster 1 (T-RF 61 bp; KJ660877), Cluster 2 Related (T-RFs 74 and 255 bp; KJ660866 and KJ660862), Cluster 3a.1 (T-RFs 69 and 70 bp; KJ660872 and KJ66087), Cluster 3b (T-RF 72 bp; KJ660883), Cluster 4 (T-RFs 46 and 103bp; KJ660867 and KJ660882), Cluster 6 (T-RF 196 bp; KJ660886) and Cluster ME (T-RFs 67, 101, 139 and 141 bp; KJ660871, KJ660889, KJ660874 and KJ660891) by sequencing and phylogenetic analysis (**Figure 3E**; Supplementary Figure S3). Most of the clones’ closest relatives were either from alpine meadow soil of Tibet (Cluster 3a.1, Cluster 4, and Cluster 6) or Mount Everest (Cluster ME and Cluster 2 associated). Of these, Cluster ME was the dominant group in the younger soils (accounted for about 60%), and then its abundance decreased to around 20% in year 44 and Ref soils. The relative abundance of species in clusters 4 and 6 were only detected in 0 year, while cluster 1 was only detected in Ref soil. *nirS, nirK* and *nosZ* genes of denitrifying bacteria also presented clear trends of succession during soil development. The genus *Pseudomonas* (T-RFs 190 and 308 bp; KJ145302 and KJ145309), *Paracoccus* (T-RF 152 bp; KJ145300) and *Sulfuritalea* (T-RF 120 bp; KJ145305) were the dominant groups in the *nirS* gene clone library (supplementary Figure S4). The proportion of the genus *Paracoccus* (α-proteobacteria) significantly increased during 17 years, with values from 17.8 to 48.6%. The genus *Pseudomonas* was the opposite in that the relative abundance decreased from 48% in 0 year to 18% in 17 years (**Figure 3G**; Supplementary Figure S5). The genus *Achromobacter* (T-RFs 53, 167, and 172 bp; KJ660950, KJ660936 and KJ660940) was the predominant group in the *nirK* gene clone library in all samples and its abundance gradually increased from 71 to 90% during 31 years, but then slightly decreased to 86% in 44 years. In the *nosZ* gene clone library, the genus *Bradyrhizobium* (T-RF 672 bp; KJ145280) was the predominant group in the 0 year soil (50%), but its relative abundance dramatically decreased during 5–17 years (about 1%), and then gradually increased from 3% in 31 years to 30% in 44 years (**Figure 3K**; Supplementary Figure S6). The uncultured species were classified into the order Rhizobiales.

The PERMAVONA test indicated that all the N cycling microbial communities significantly changed over time (*p* < 0.05). The Principal coordinate analysis (PCoA) showed that the shift in N cycling microbial community composition was structured by successional age (**Figure 3**). The Shannon-wiener index of *nifH*, AOA-*amoA, nirK/S* containing communities significantly decreased with successional time, while AOB-*amoA* and *nosZ* containing communities were the opposite.

### The Driving Factors of the Changes in N Cycling Microbial Community

**FIGURE 4:**
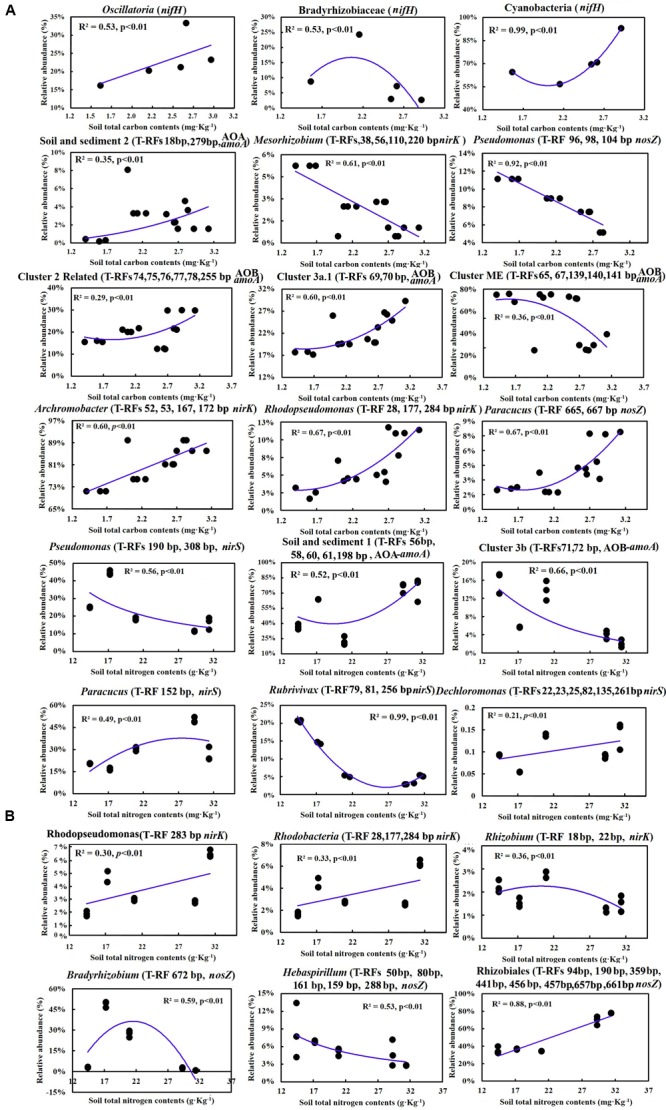
**(A)** Correlations between soil total C and the relative abundance of major groups in different steps of the N cycling microbial community; **(B)** Correlations between soil total N and the relative abundance of major groups in different steps of the N cycling microbial community.

Multiple linear regression analysis revealed a positive and significant influence of soil TC on *nifH*, AOB-*amoA, nirK/S* and *nosZ* genes. Soil age was only significantly related to AOA-*amoA* gene copies (**Table 3**). The Mantel test indicated that TC and TN contents were consistently and significantly correlated with all the N cycling microbial communities (*p* < 0.05) (**Table 4**). Of these, N cycling microbial communities that were dominant in younger soils (0–5 years) were significantly correlated with TC (**Figure 4A**), while communities that were most abundant in older soils (17 years to Ref) were significantly correlated with TN (**Figure 4B**). Soil age was another important factor that was significantly and positively correlated with AOA, AOB, *nifH* and *nosZ*-containing communities (*p* < 0.001). AP was positively correlated with the *nifH*-containing community (*p* < 0.01) (**Table 4**). None of the soil parameters were consistently correlated with all N cycling microbial communities’ diversity. TC and soil age were significantly correlated with AOA, *nosZ* and *nifH*-containing communities. TN was significantly correlated with AOB, *nirK, nosZ* and *nifH*-containing communities. AOA (*r* = 0.56, *p* < 0.01) was significantly but negatively correlated with soil age, whereas *nosZ*-containing communities (*r* = 0.88, *p* < 0.01) was significantly and positively correlated with soil age.

**Table 3:** Multiple regression analysis between functional gene level and TC, TN, and successional age as independent factors.

Parameters	Standardized coefficient (β)^a^			Model *P* value
	TC	TN	Age	
*nifH* level	0.82^∗∗^	0.085	0.018	0.00
AOA level	-0.40	-0.55^∗∗^	0.57^∗∗^	0.001
AOB level	0.81^∗∗^	0.067	0.056	0.001
*nirS* level	0.74^∗∗^	0.48^∗∗^	0.39	0.01
*nirK* level	0.56^∗^	0.04	-0.85	0.001
*nosZ* level	0.74^∗∗^	0.78^∗∗^	0.41	0.01

*^*a*^Significance levels (*p*) of the multiple regression model are as follows: ^∗^*p*< 0.05; ^∗∗^*p* < 0.01.*

**Table 4:** Mantel correlations between the microbial community structure and soil characteristics.

Soil parameters	AOA	AOB	*nirS*	*nirK*	*nosZ*	*nifH*
TC (g⋅kg^-1^)	0.36^∗∗^	0.41^∗∗^	0.34^∗∗^	0.27^∗^	0.32^∗∗^	0.25^∗^
TN (g⋅kg^-1^)	0.43^∗∗^	0.20^∗^	0.56^∗∗^	0.71^∗∗^	0.61^∗∗^	0.32^∗∗^
Age (years)	0.40^∗∗^	0.80^∗∗^	0.10	0.090	0.40^∗∗^	0.38^∗∗^
TP (g⋅kg^-1^)	-0.061	0.37	-0.037	-0.11	-0.11	-0.0034
AP (mg⋅kg^-1^)	0.17	0.31	-0.028	0.094	0.28^∗^	0.52^∗∗^
NH_4_^+^ (mg⋅kg^-1^)	0.22	0.84^∗∗^	0.096	0.010	0.042	0.38^∗∗^
NO_3_^-^ (mg⋅kg^-1^)	-0.028	0.31^∗∗^	0.075	0.029	-0.051	0.35^∗∗^
pH	-0.038	0.26	0.20	-0.083	-0.11	0.016

*Significance levels (*p*): ^∗^*p* < 0.05; ^∗∗^*p* < 0.01.*

## Discussion

### Succession of N Cycling Microbial Communities Across the Glacier Foreland

We found that N cycling functional gene abundances increased with time, which was in agreement with previous research in Tianshan Mountain No.1 glacier, showing that N cycling functional gene abundances increased along the chronosequence (Segawa et al., 2014). However, Brankatschk et al. (2011) showed that ammonia oxidizing microbial community abundances increased with time, but denitrifying bacterial (*nirK/S* and *nosZ*) gene abundances decreased in the Damma Glacier forefield. Similar results were found in Rotmoosferner glacier foreland (Ötz Valley, Austria) which showed denitrifying community gene abundances decreased with time (Kandeler et al., 2006). One possible reason was that the appearance of plant patches in older soils may reduce the relative availability of ammonia and thus influence denitrifying bacterial abundance (Brankatschk et al., 2011). Further community composition analysis of results showed that N cycling microbial community compositions also significantly changed during 44 years and were structured by successional age (**Figure 3**). Similar results were observedfor N_2_-fixing bacteria and denitrifying bacteria in Alpine glaciers (Tscherko et al., 2004; Deiglmayr et al., 2006; Nemergut et al., 2007; Duc et al., 2009).

Our findings provided support for the hypothesis that N cycling processes were initially started from diazotrophs at the initial sites of the glacier foreland (Ollivier et al., 2011), as evidenced by the predominance of the heterocystous cyanobacteria (e.g., *Anabaena, Nostoc* and *Cylindrospermum*) in the initial soils (0 and 5 years), which can directly transform N_2_ into ammonium as free-living or associated with plant roots (Duc et al., 2009). Ollivier et al. (2011) argued that due to the poor nutrition and low N levels in newly exposed barren soils, N input is not likely to be derived from the weathering of bedrock material or highly energy demanding processes (like mineralization of recalcitrant materials). Hence, autotrophic N_2_-fixing bacteria seem to be the major pathway for the initial N input in soils. The amount of ammonia derived from the N_2_ fixation process, although low in concentration, is sufficient to activate the nitrification process and significantly influence the development of microbial communities involved in nitrification in the glacier as proposed by Ollivier et al. (2011).

Segawa et al. (2014) found isotope ratio evidence of the occurrence of nitrification on the surface of the Tianshan Mountain No.1 glacier. Past research indicated that in the glacier foreland ecosystem the ammonia-oxidizing process was mainly driven by AOA, due to the AOA having a better adaptability to the ammonia-poor environment and lower pH than AOB (Di et al., 2009; Ollivier et al., 2011). On the contrary, we speculated that the ammonia-oxidizing process was mainly driven by AOB, as evidenced by the weakly alkaline soil pH. A large number of studies have shown that AOA was the main contributor to nitrification in acid soils and their nitrification activity decreased with increasing pH (Shen et al., 2008). Besides, only AOB-*amoA* gene abundance and community composition showed a significant correlation with NH_4_^+^ concentration.

Community composition analysis showed the *Nitrosospira* cluster ME (AOB community first discovered and designated in Mount Everest soils at altitude ≥5700 m a.s.l) and the *Nitrososphaera* cluster (AOA community dominant in Mount Everest soils at altitude ≥6100 m a.s.l) were the dominant groups in younger soils. Their dominance was replaced by the *Nitrosospira* cluster 3 (≤5400 m a.s.l) of AOB and Soil and sediment 1 cluster (≤5800 m a.s.l) of AOA, respectively, in older soils. Zhang et al. (2009) and other researchers believed that the AOA and AOB composition shift between lower and higher altitudes was mainly attributed to the variation of temperature (Avrahami and Conrad, 2003). In the present study, the initial stage sampling sites were close to the glacier terminus so that soil temperature was lower, but as sampling distance increased, soils tended to be exposed to sunlight with patches of plants, and thus soil temperatures were higher. Our hypothesis was further supported by the group clusters 4 and 6 (AOB community) which were detected in 0 year, and were only detected in the cold temperate soils (Avrahami and Conrad, 2003, 2005).

In recent years, it was discovered that some bacteria can carry out aerobic denitrification, and even some can perform heterotrophic nitrification simultaneously. One typical example is the bacterium *Paracoccus pantotrophus*, which is capable of heterotrophic nitrification-aerobic denitrification (Robertson and Kuenen, 1983). In the present study, we found that nearly all the denitrifying clones’ closest relatives were potential aerobic denitrifying bacteria, such as *Paracoccus pantotrophus, Achromobacter xylosoxydans, Pseudomonas putida, Achromobacter xylosoxydans, Rhodococcus stutzeri*, and *Alcaligenes faecalis* in all soil samples (Joo et al., 2005; Chen et al., 2012; Kundu et al., 2012). Besides, some other denitrifying processes involved microorganisms were also found, such as autotrophic nitrate-reducing sulfide-oxidizing bacteria (NR-SOB) and denitrifying phosphorus removing bacteria (DPB). For instance, nearly 20% of clones showed high sequence similarity to a novel facultative autotrophic bacterium *Sulfuritalea hydrogenivorans*, which can oxidize thiosulfate and utilize nitrate as an electron acceptor (Kojima and Fukui, 2011). In the present study, its highest abundance was in 5 years (accounted for 30%) and its abundance was not obviously changed in other samples (around 20%). DPB species in the genera *Accumulibacter* and *Dechloromonas* were detected in all soil samples and they presented a clear increasing trend along the stages. These kinds of species could accumulate soluble orthophosphate under anaerobic conditions and utilize nitrate as an electron acceptor in oxygen–deficient conditions. Therefore, the mechanism of denitrification in the glacier foreland soils deserves further investigation.

### The Succession of N Microbial Community Composition and Diversity as Driven by Soil C and N

Spearman correlation analysis showed that the shift of the most abundant functional communities along the chronosequence was mainly attributed to the dynamic changes of TC and TN. The results suggested that soil C and N contents drove the shift of functional communities. The observations were in agreement with previous studies, which indicated that soil C and N were the most limiting resources that influenced soil development and soil microbial community composition in primitive soils (Sigler and Zeyer, 2002; Tscherko et al., 2003, 2004; Göransson et al., 2011). Sigler and Zeyer (2002) attributed the dynamic shift of microbial community composition to the different adaptation strategies in different stages. For example, microbial organisms undergo an R-K strategy as macroorganisms, and the potential explanation was usually ascribed to the shift of soil C and N availability in different stages. Wardle et al. (2004) showed that soil C was the most limiting factor for microbial growth and activity in early, barren soils, and the N-limitation effect obviously occurred in older, vegetated soils. With increasing soil C the N limitation to bacterial growth became more apparent, suggesting that microbial communities in older soils were more affected by soil N content (Göransson et al., 2011).

Carbon availability usually decreases with the accumulation of soil C, because it will aggregate into recalcitrant complexes with time. Hence, the decline of C availability usually leads to a decline of microbial diversity, which was evidenced by previous work at the Damma glacier forefield. Not only did bacterial diversity decline, but also N_2_ fixing and denitrifying bacterial diversity declined with successional time (Sigler and Zeyer, 2002; Deiglmayr et al., 2006; Duc et al., 2009). Our results are in agreement with those observations, showing a significant decline of *nifH* and AOA diversity, as well as a decline of *nirS* and *nirK* (**Table 3**). In younger soils, the low population density and lack of vegetation leads to limited competition, although soil C availability was relatively high. With vegetation establishment in older soils (17–44 years), plants and microorganisms compete for the C, N, and P resources, thus leading to a decrease in diversity (Tscherko et al., 2004; Brankatschk et al., 2011). Moreover, in the developed stage, the rhizosphere effect and selection to the soil microbial community appeared and resulted in decreased diversity, despite microbial community abundance being significantly increased (Duc et al., 2009). Therefore, in the present study, the family Nostocaceae was positively correlated with TC. Species in the genus *Nostoc* could not only be free-living and fixing N_2_ by heterocyst, but were also able to form both loose and tight association with the roots of gramineous plants (Gantar et al., 1991). Besides, species in the families Opitutaceae, Chromatiaceae and Methylobacteriaceae were only detected in the developed stage, and were significantly correlated with TC, which indicated that they may rely on root exudates to fuel heterotrophic N_2_ fixation (Hamelin et al., 2002).

## Conclusion

Our research indicated that the soil N cycling microbial communities either expressed as gene abundances or composition were significantly changed during 44 years. Soil TC was the main driving factor that caused the shift of the N cycling microbial community gene abundances. N cycling microbial communities was structured by successional age, and underwent predictable changes through time. TC, TN and soil age were the most important factors that explained the most variance of N cycling microbial communities Besides, ammonia oxidizing archaea and bacteria in the glacial foreland soil were more likely to be cold tolerant, and thus further studies are needed to reveal their mechanism. Bacterial species potentially capable of heterotrophic nitrification-aerobic denitrification were dominant in the glacial soils; hence to a better understanding of N cycling in the ecosystem, it will be interesting to make comparisons between autotrophic nitrification and heterotrophic nitrification-aerobic denitrification processes and their contribution to the N cycling related to N_2_O or N_2_ fluxes.

## Author Contributions

JZ, KL, J-ZH designed this study. JZ, KL, C-JZ, J-TW performed the laboratory analysis. All the authors attended data analyzing and manuscript writing.

## Conflict of Interest Statement

The authors declare that the research was conducted in the absence of any commercial or financial relationships that could be construed as a potential conflict of interest.
