# Supplementary material for: Primary Succession of Nitrogen Cycling Microbial Communities Along the Deglaciated Forelands of Tianshan Mountain, China
Source: Front Microbiol. 2016 Aug 30;7:1353. doi: 10.3389/fmicb.2016.01353 (PMC5003921; doi:10.3389/fmicb.2016.01353)
Supplement: Supplementary file 1 [file Data_Sheet_1.DOCX]

**Supplementary figure legends**

**Figure s1.** Neighbor-joining phylogenetic tree based on nitrogen-fixing (*nifH*) gene clone library from forelands of the Tianshan Mountains Glacier No. 1, numbers on the nodes are the bootstrap values (percentages) based on 1,000 replicates and values of above 50% were presented.

**Figure s2.** Neighbor-joining phylogenetic tree based on ammonia-oxidizing archaeal (AOA) *amoA* gene clone library from forelands of the Tianshan Mountains Glacier No. 1, numbers on the nodes are the bootstrap values (percentages) based on 1,000 replicates and values of above 50% were presented. The sequences identified for the TRFs digested by the *Rsa* I enzyme are highlighted in blue.

**Figure s3.** Neighbor-joining phylogenetic tree based on ammonia-oxidizing bacterial (AOB) *amoA* gene clone library from forelands of the Tianshan Mountains Glacier No. 1, numbers on the nodes are the bootstrap values (percentages) based on 1,000 replicates and values of above 50% were presented. The sequences identified for the TRFs digested by the *Hha* I enzyme are highlighted in blue.

**Figure s4.** Neighbor-joining phylogenetic tree based on nitrite reductases gene *nirS* clone library from forelands of the Tianshan Mountains Glacier No. 1, numbers on the nodes are the bootstrap values (percentages) based on 1,000 replicates and values of above 50% were presented. The sequences identified for the TRFs digested by the *Hae* III enzyme are highlighted in blue.

**Figure s5.** Neighbor-joining phylogenetic tree based on nitrite reductases coding gene *nirK* clone library from forelands of the Tianshan Mountains Glacier No. 1, numbers on the nodes are the bootstrap values (percentages) based on 1,000 replicates and values of above 50% were presented. The sequences identified for the TRFs digested by the *Hae* III enzyme are highlighted in blue.

**Figure s6.** Neighbor-joining phylogenetic tree based on nitrous oxide coding gene *nosZ* clone library from forelands of the Tianshan Mountains Glacier No. 1, numbers on the nodes are the bootstrap values (percentages) based on 1,000 replicates and values of above 50% were presented. The sequences identified for the TRFs digested by the *Rsa* I enzyme are highlighted in blue.
