# Supplementary figures and images for: Primary Succession of Nitrogen Cycling Microbial Communities Along the Deglaciated Forelands of Tianshan Mountain, China
Source: Front Microbiol. 2016 Aug 30;7:1353. doi: 10.3389/fmicb.2016.01353 (PMC5003921; doi:10.3389/fmicb.2016.01353)

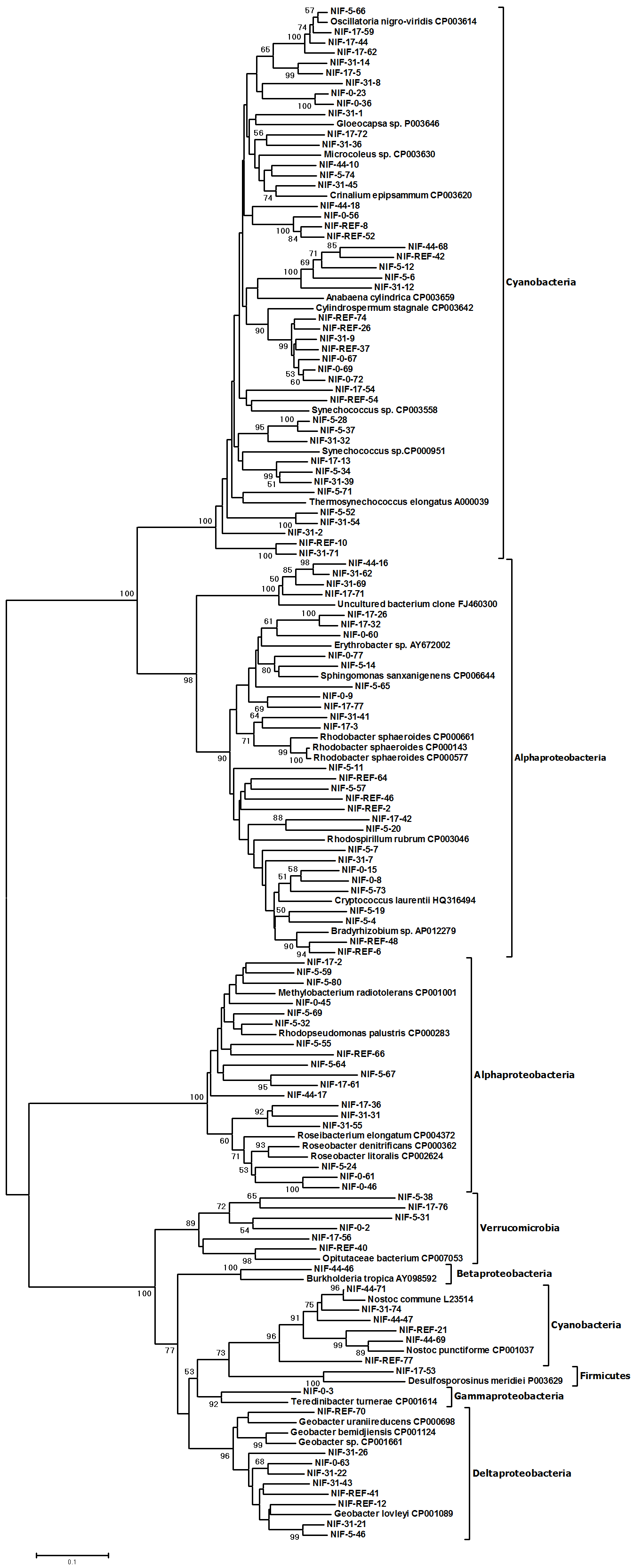

Supplement: Supplementary file 2 [file Image_1.TIF]

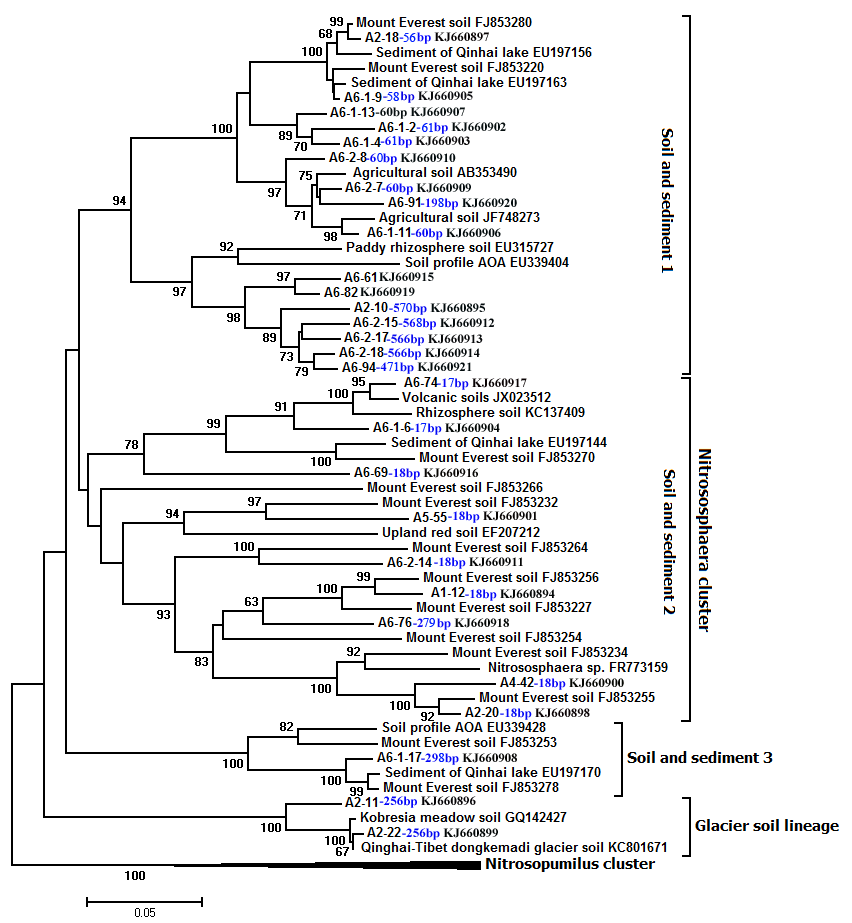

Supplement: Supplementary file 3 [file Image_2.TIF]

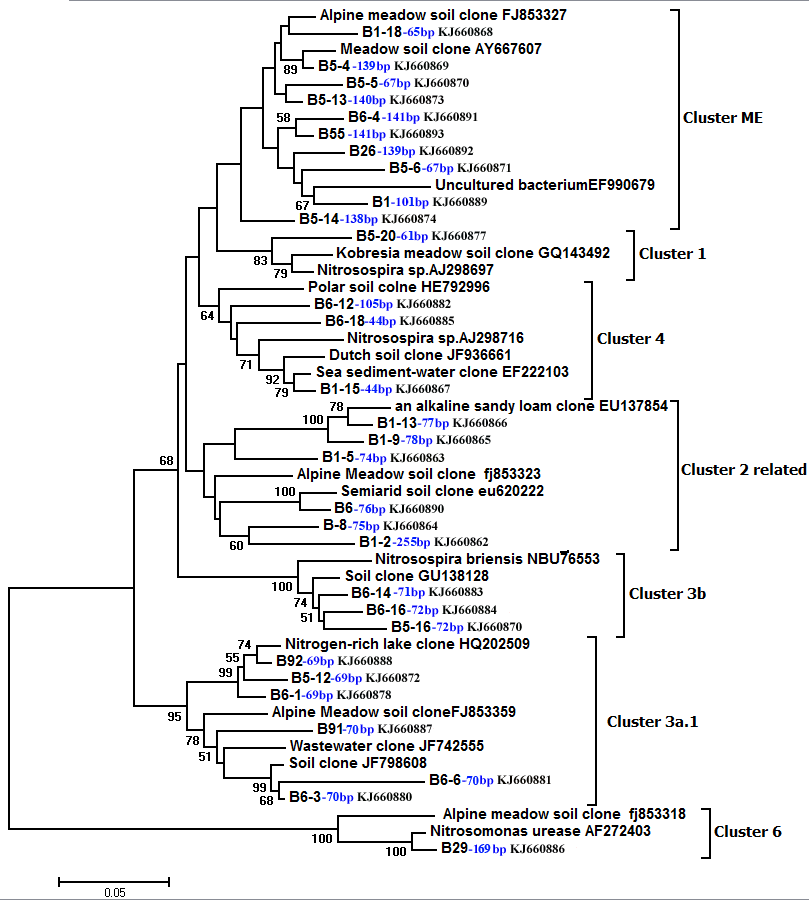

Supplement: Supplementary file 4 [file Image_3.TIF]

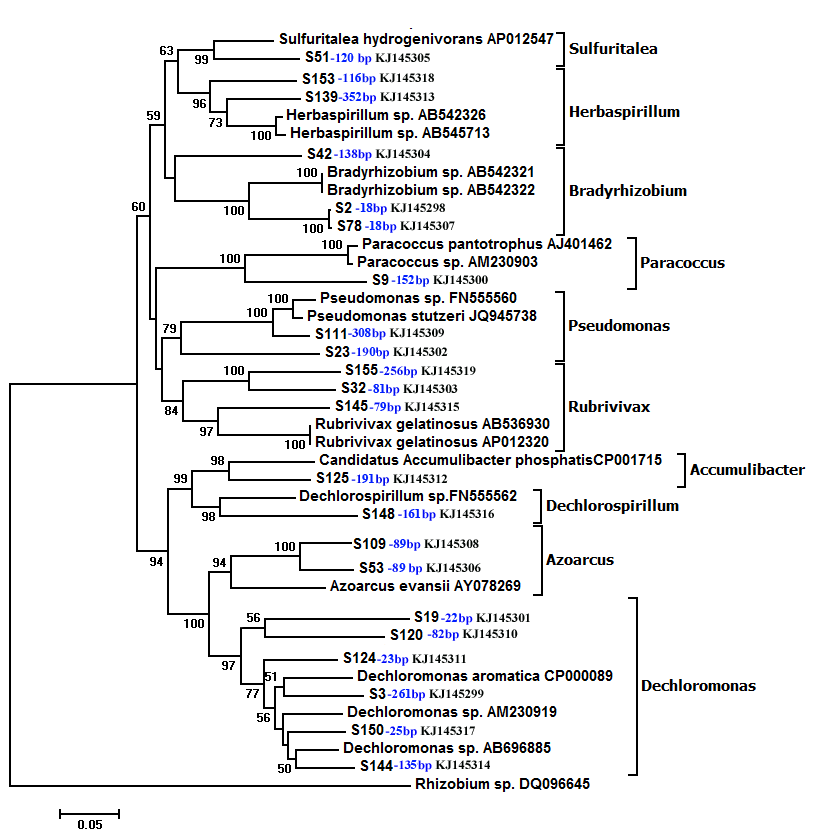

Supplement: Supplementary file 5 [file Image_4.TIF]

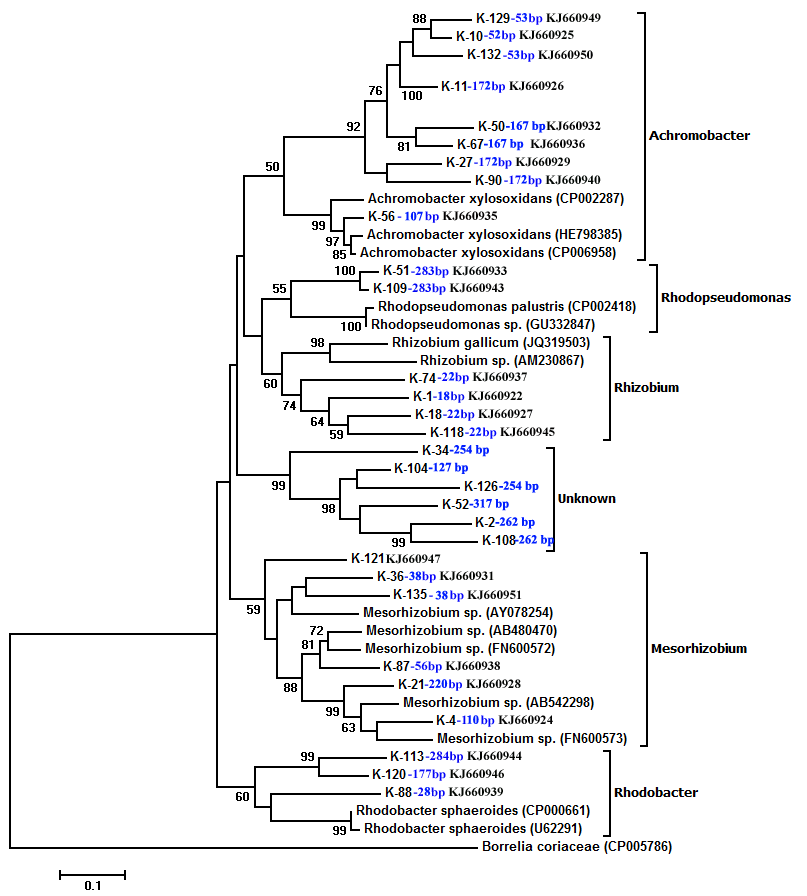

Supplement: Supplementary file 6 [file Image_5.TIF]

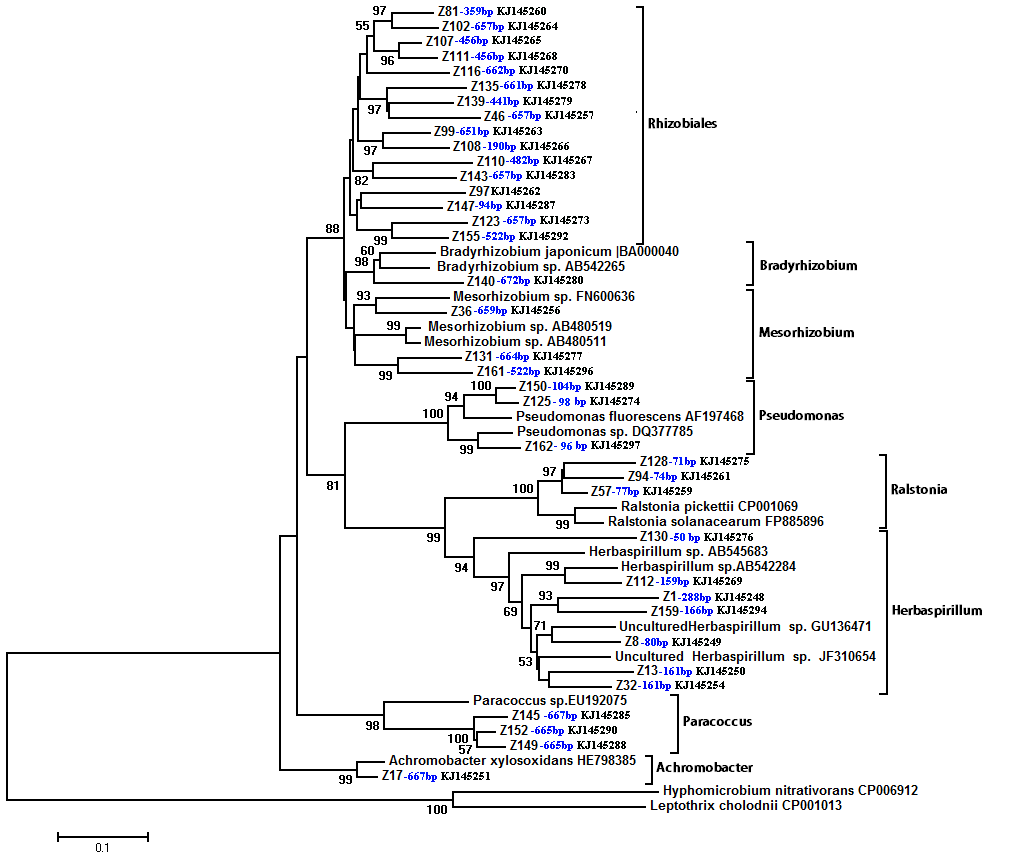

Supplement: Supplementary file 7 [file Image_6.TIF]
